# Beyond survey design: Lessons from conducting the National Adolescent Mental Health Surveys

**DOI:** 10.1186/s13034-025-00925-1

**Published:** 2025-07-31

**Authors:** Holly E. Erskine, Yohannes Dibaba Wado, Vu Manh Loi, Dao Thi Khanh Hoa, Amirah Ellyza Wahdi, Mengmeng Li, James G. Scott

**Affiliations:** 1 School of Public Health, The University of Queensland, Herston, QLD Australia; 2 Queensland Centre for Mental Health Research, Wacol, QLD Australia; 3 Institute for Health Metrics and Evaluation, University of Washington, Seattle, WA USA; 4 African Population and Health Research Center, Nairobi, Kenya; 5 Institute of Sociology, Vietnam Academy of Social Sciences, Hanoi, Vietnam; 6 Tradition and Development Research Institute, Hanoi, Vietnam; 7 Center for Reproductive Health, Faculty of Medicine, Public Health, and Nursing, Universitas Gadjah Mada, Yogyakarta, Indonesia; 8 Department of Biostatistics, Epidemiology, and Population Health, Faculty of Medicine, Public Health, and Nursing, Universitas Gadjah Mada, Yogyakarta, Indonesia; 9 Department of Population, Family and Reproductive Health, Johns Hopkins Bloomberg School of Public Health, Johns Hopkins University, Baltimore, MD USA; 10 Child Health Research Centre, The University of Queensland, South Brisbane, QLD Australia; 11 Child and Youth Mental Health Service, Children’s Health Queensland, South Brisbane, QLD Australia

**Keywords:** Mental health, Adolescent, Low- and middle-income countries, Survey, Design, Compromise, Communication, Collaboration

## Abstract

**Background:**

The National Adolescent Mental Health Surveys (NAMHS) were the result of a six-year collaboration between five organisations from five countries. Nationally representative household surveys of adolescents aged 10–17 years and their primary caregiver were conducted in 2021 in Kenya, Indonesia, and Vietnam.

**Compromise and communication:**

Despite challenges, including the global COVID-19 pandemic, NAMHS was able to produce high-quality data which are featured in this Supplement. The operationalisation of compromise and communication were key factors in navigating the complexity of conducting three parallel surveys while also incorporating the knowledge and expertise of the teams from all five organisations. Compromise was an ongoing feature of NAMHS, including in relation to the choice of measures as well as their administration. Effective communication was realised through a comprehensive system that was implemented from the inception of NAMHS, ensuring meaningful and effective communication between the five teams for the benefit of all three surveys. The approach to compromise and communication was a considerable factor in the ability of NAMHS to not only weather the COVID-19 pandemic but also improve the project during the subsequent delays to data collection.

**Conclusion:**

While factors such as compromise and communication are generally central to successful research collaborations, they are rarely mentioned in survey methodology. Future collaborations undertaking complex cross-national research would greatly benefit from taking a proactive and planned approach to communication and compromise.

## Background

The National Adolescent Mental Health Surveys (NAMHS) measured the prevalence of mental disorders, as well as risk and protective factors and service use, among adolescents aged 10–17 years in Kenya, Indonesia, and Vietnam through nationally representative household surveys [1, 2]. Five organisations from five countries worked together over a six-year cross-national research collaboration: The University of Queensland (UQ), Australia (lead of NAMHS); the African Population and Health Research Center, Kenya (lead of K-NAMHS); Universitas Gadjah Mada, Indonesia (lead of I-NAMHS); the Institute of Sociology, Vietnam (lead of V-NAMHS); and the Johns Hopkins Bloomberg School of Public Health, USA (collaborating partner). NAMHS found that the prevalence of any mental disorder in the past 12 months differed significantly by country, with further variation seen at the individual disorder level [1]. In addition to generating data for adolescent mental health that was previously not available in these countries or surrounding regions, the methodology of NAMHS itself is also an important contribution to the wider literature [3], with substantial efforts made to ensure the cross-national comparability of the data balanced with the need to ensure the relevance of the data in country [1].

The development of NAMHS by teams within these five organisations was driven by core principles of collaboration, cultural relevancy, comparability, capacity building, and translation of findings [4], in addition to drawing upon best practice for survey design and prioritising data quality and research integrity. These original principles were further operationalised through embracing compromise and establishing a comprehensive communication system. These aspects of research management, rarely reported in descriptions of survey methods, were crucial to the ability of NAMHS to overcome unforeseen challenges, including the COVID-19 pandemic, while producing the high-quality data featured in this Supplement.

## Compromise

The development and adaptation of a single instrument to suit parallel surveys in three different countries, while also incorporating the perspectives and expertise of five teams, necessitated ongoing constructive compromise. The instrument underwent translation and back-translation, clinical review by in-country clinicians, and several rounds of revision, with decisions made collaboratively across all five teams to ensure conceptual consistency across all three countries [1]. Compromises were made from the choice of measures through to their adaptation and implementation, while also further considering potential future use of NAMHS data and methodology. A consequence of this approach was potentially fewer country-specific modules due to priority being given to modules/questions that allowed for meaningful cross-national comparison. Wherever possible, country-specific considerations were incorporated e.g., the inclusion of a problematic internet use and electronic gaming in Kenya, and the inclusion of country-specific terms to describe different substances [1].

The Diagnostic Interview Schedule for Children, Version 5 (DISC-5) [5, 6] was chosen as the primary measure of adolescent mental disorders in NAMHS. The DISC-5 generates mental disorder diagnoses according to established diagnostic criteria [7], with these recognised by international monitoring agencies, in-country Ministries of Health, and the Global Burden of Disease Study as per the requirements for data to be included in the quantification of disease burden [8]. While briefer diagnostic measures of mental disorders were available, the comprehensiveness of the DISC-5 provided the capacity for future investigation of culturally informed adjustments to scoring algorithms, alternate symptom weighting, and patterns of symptoms and impairment among those with subthreshold mental disorders. This capacity is evidenced in the studies within this Supplement, with respective analyses including diagnostic [9–11] and subthreshold [12, 13] mental disorders as appropriate to their research questions. In addition, the DISC-5 is free to use with permission of the DISC developer. Again, while other diagnostic measures were available, the associated cost would have been prohibitive to NAMHS as well as any future efforts to replicate the methodology. The consideration of cost was also reflect in the programming of the instrument, where no- to low- cost options with proven efficacy in low resource settings were used [14, 15] instead of more expensive options.

The approach to compromise continued through to the administration of the measures themselves. For example, although the NAMHS instrument was originally intended to be completely interviewer-administered, three modules were changed to self-administration– sexual behaviour, adverse childhood experiences, and substance use– in response to feedback from the in-country NAMHS teams [2] and previous evidence of reporting bias for sensitive issues [16, 17]. In addition, the sexual behaviour module, inclusive of sexuality and gender identity questions [12], was only asked to adolescents aged 12 years and older. While previous studies have assessed sexual health and behaviour among younger adolescents [18, 19], the age restriction applied in NAMHS was a compromise to ensure all adolescents were more likely to understand the questions (which did not have the scope to go into more detail or come with additional explanation) and that there was less chance of any unintended adverse impact on parental consent to participate in the survey.

## Communication

The approach to compromise was facilitated through a comprehensive system of communication, established from the inception of NAMHS. A key feature of this was enabling and encouraging communication between all five teams, not just bilateral communication between the lead UQ team and each respective NAMHS team. For example, online meetings involving all five teams happened on a regular basis (from weekly to monthly depending on the project phase), in addition to weekly online meetings between the lead UQ team and each NAMHS team. These meetings were designed not only as a forum for status updates but also to specifically to facilitate discussion amongst all teams, with all individuals able to raise issues and ideas, and to add items to meeting agendas. This was supplemented by three in-person meetings of all five teams (2018, 2020, 2022), in-country planning meetings (2019), and in-country trainings (2019). Further, all scheduling accounted for the national and religious holidays in each country, reflecting cultural sensitivity in the conduct of NAMHS as well as the survey instrument itself.

For every meeting, all discussions were recorded and converted into decisions and action items which were circulated between all teams. This system served as the ‘lifeblood’ of NAMHS, enabling the lead UQ team to ensure all issues were identified, tracked, and resolved across a complex and evolving project, while also allowing information and improvements to be communicated and applied across all three surveys and between all five organisations. The approach to communication continued post data collection, i.e., throughout data cleaning and the finalisation of all three survey datasets, ensuring that the consistency achieved during data collection was also reflected in the final outputs of each survey. While initially it may seem obvious that such a communication system would be necessary in an undertaking such as NAMHS, these strategies are rarely detailed (if even mentioned) in descriptions of survey methodology and the study would potentially have further benefitted from this approach being incorporated from the outset rather than evolving organically during survey development.

## COVID-19

The approach to compromise and communication was a key factor in overcoming the unprecedented challenge of the global COVID-19 pandemic. The pre-existing communication system already in place enabled the NAMHS teams to adapt quickly and cooperatively to the ever-evolving situation, particularly as it became clear that the original planned commencement of data collection in March (Kenya and Indonesia) and June (Vietnam) 2020 was no longer feasible. It also meant that a new system of management and communication did not have to be created and implemented during an already challenging period. In addition, the communication processes already in place allowed the NAMHS teams to collectively take full advantage of the unanticipated 12- to 15-month delay to data collection. This included substantial revision of the NAMHS instrument, comprehensive testing of the programming in relation to both the instrument and survey processes, and updates to fieldwork processes to include COVID-19-safe protocols. Further, all five teams worked together to develop a COVID-19 module that was included in the final NAMHS instrument, again compromising to include questions relevant both in-country and cross-nationally. This allows for future analyses to investigate the potential impact of COVID-19 on the prevalence of adolescent mental disorders reported by NAMHS and compare the extent and nature of this impact consistently among the three countries.

## Conclusions

In addition to the vital contribution of the data itself, NAMHS serves as a ‘blueprint’ for future research, providing guidance on how to conduct cross-national mental health surveys among young people in LMICs as well as how to do so in a genuinely collaborative manner for the benefit of the study and the individuals involved. While the methodology of NAMHS was based on best practice in survey methodology, it was the approach to compromise and communication that enabled NAMHS to be successfully conducted in three countries in parallel during unforeseen global circumstances. Further, an unplanned yet pivotal advantage of this approach was the resulting strong research collaboration which remains active and productive despite NAMHS having been completed. Future research collaborations, particularly those occurring across different countries and cultures, would benefit from proactively and authentically incorporating these approaches into core survey methodology and research management.

## Data Availability

The NAMHS datasets are co-owned by the University of Queensland (UQ) and each respective in-country lead organisation (K-NAMHS: African Population and Health Research Center [APHRC] and UQ; I-NAMHS: UGM and UQ; V-NAMHS: Institute of Sociology [IOS] and UQ). Currently, these datasets or analysis of these datasets are available for collaborative work on request to the relevant data owners following an established protocol. Work is currently underway to convert the NAMHS datasets into public use datasets, allowing for wide use of these datasets while ensuring protection of participant privacy, adherence to country-specific legislation, and appropriate use of data. This includes development of accompanying meta-data, inclusive of a codebook, technical manual, and analysis files. The expected launch date for these public use datasets and accompanying meta-data is 2026, with hosting mechanisms currently under development in line with country legislation and ethical requirements.

## Abbreviations

DISC-5: Diagnostic Interview Schedule for Children, Version 5
I-NAMHS: Indonesia–National Adolescent Mental Health Survey
K-NAMHS: Kenya–National Adolescent Mental Health Survey
NAMHS: National Adolescent Mental Health Surveys
UQ: The University of Queensland
V-NAMHS: Viet Nam Adolescent Mental Health Survey
